# Grey literature in systematic reviews: a cross-sectional study of the contribution of non-English reports, unpublished studies and dissertations to the results of meta-analyses in child-relevant reviews

**DOI:** 10.1186/s12874-017-0347-z

**Published:** 2017-04-19

**Authors:** Lisa Hartling, Robin Featherstone, Megan Nuspl, Kassi Shave, Donna M. Dryden, Ben Vandermeer

**Affiliations:** 1 Cochrane Child Health, University of Alberta, ECHA4-472, 11405-87 Avenue, Edmonton, AB T6G 1C9 Canada; 2 Alberta Research Center for Health Evidence (ARCHE), University of Alberta, 11405-87 Avenue, Edmonton, AB T6G 1C9 Canada

**Keywords:** Systematic reviews, Literature searching, Meta-analysis, Knowledge synthesis, Publication bias, Language bias, Grey literature, Unpublished studies, Dissertations, Non-English publications

## Abstract

**Background:**

Systematic reviews (SRs) are an important source of information about healthcare interventions. A key component of a well-conducted SR is a comprehensive literature search. There is limited evidence on the contribution of non-English reports, unpublished studies, and dissertations and their impact on results of meta-analyses.

**Methods:**

Our sample included SRs from three Cochrane Review Groups: Acute Respiratory Infections (ARI), Infectious Diseases (ID), Developmental Psychosocial and Learning Problems (DPLP) (*n* = 129). Outcomes included: 1) proportion of reviews that searched for and included each study type; 2) proportion of relevant studies represented by each study type; and 3) impact on results and conclusions of the primary meta-analysis for each study type.

**Results:**

Most SRs searched for non-English studies; however, these were included in only 12% of reviews and represented less than 5% of included studies. There was a change in results in only four reviews (total sample = 129); in two cases the change did not have an impact on the statistical or clinical significance of results. Most SRs searched for unpublished studies but the majority did not include these (only 6%) and they represented 2% of included studies. In most cases the impact of including unpublished studies was small; a substantial impact was observed in one case that relied solely on unpublished data. Few reviews in ARI (9%) and ID (3%) searched for dissertations compared to 65% in DPLP. Overall, dissertations were included in only nine SRs and represented less than 2% of included studies. In the majority of cases the change in results was negligible or small; in the case where a large change was noted, the estimate was more conservative without dissertations.

**Conclusions:**

The majority of SRs searched for non-English and unpublished studies; however, these represented a small proportion of included studies and rarely impacted the results and conclusions of the review. Inclusion of these study types may have an impact in situations where there are few relevant studies, or where there are questionable vested interests in the published literature. We found substantial variation in whether SRs searched for dissertations; in most reviews that included dissertations, these had little impact on results.

**Electronic supplementary material:**

The online version of this article (doi:10.1186/s12874-017-0347-z) contains supplementary material, which is available to authorized users.

## Background

Knowledge syntheses, including systematic reviews (SRs), provide essential evidence to inform healthcare decision making [1]. A key component of a well-conducted SR is an objective, sensitive, and reproducible literature search of multiple sources [2]. Methodological standards for SRs recommend extensive searching to address the potential for publication bias, to reflect the totality of evidence on a given question and produce accurate and valid estimates of effect [3–6]. Current Cochrane guidance recommends searching in grey literature sources beyond conventional bibliographic biomedical databases (e.g., Medline or Embase) for unpublished data, including trials registries, government regulatory documents and conference proceedings [4, 7]. The Institute of Medicine and the Agency for Healthcare Research and Quality (AHRQ) also recommend (in addition to electronic databases) searching grey literature databases, clinical trials registries and other sources of unpublished information [5, 6]. However, there is limited empiric evidence about the potential impact of selective searching and inclusion on the results of SRs.

Direct and indirect evidence confirm that studies which report large effect sizes or findings of beneficial interventions are published more frequently [3]. The impact of unpublished trials on the results of 60 meta-analyses on different medical interventions found that unpublished trials were less likely to produce statistically significant or beneficial results compared with published trials [8]. In most instances, the exclusion of these unpublished trials in meta-analyses had relatively small impact on estimates of treatment effectiveness and the changes in effect were inconsistent [8]. Other methods research has also observed the contribution of unpublished studies to reduce or change effect estimates of SRs [9–12] and to expose underestimates of harms in published studies [10, 12, 13].

Dissertations and theses are also recognized as sources of study data that, while published by academic institutions who award degrees, are not routinely published in commercial journals or indexed in conventional bibliographic databases [14]. As a sub-category of grey literature, dissertations may report studies of relevance to SR producers. However, we are unaware of investigations that have attempted to quantify the contributions of dissertations and theses to SR results.

Language bias is also a concern for systematic reviewers, [15] although empiric evidence of the existence and direction of a bias is inconsistent when non-English language publications are excluded [6]. Research suggests that German investigators are more likely to publish positive trial results in English-language publications [16, 17]. But contrary evidence from a study of acupuncture trials found that researchers from some countries (e.g., China, Russia) were more likely to publish positive findings in non-English publications [18]. Research suggests that language bias in trials is variable by topic, and that SRs of complementary and alternative medicine (CAM) interventions are more likely to be significantly impacted by the exclusion of non-English studies [19]. An investigation of SRs on herbal medicines found that relevant Chinese studies would have been missed if reviewers included only Medline-indexed journal articles [20]. Non-English language trials were also found to be prevalent in fields of psychiatry, rheumatology and orthopaedics; but SRs of exclusively English trials in these areas were found to produce similar results to reviews with no language restrictions [8]. Regardless of the impact of language bias, Cochrane guidance supports identification and inclusion of trials in all languages [2, 4] while the Institute of Medicine recommends searching in languages other than English if appropriate for the review topic [5].

Comprehensive literature searching is widely recommended; however, searching additional sources is associated with diminishing returns, [21] and the effect of continuing to search new sources has an unknown impact on the final results and conclusions. Further, inclusion of non-English studies can add substantially to the resources required to complete SRs. Given the existing environment of limited resources and the push for increased efficiencies, particularly in the context of decision-makers who require quicker production of reviews to meet their needs, [22] limits on the number of sources searched and types of studies included are necessary. The objective of this study was to examine the impact of searching for and including non-English studies, unpublished studies, and dissertations on the results of existing SRs.

## Methods

This paper reports on additional analyses that were conducted as part of a broader methodological research initiative to generate empiric evidence about the impact of searching on the results of SRs. The methods have been described in detail previously [23].

### Sample

We derived our sample from the Cochrane Child Health register of SRs which is organized in a REDCap database. The rationale for choosing reviews from the CDSR for this analysis is provided in our previous publication [23]. We exported all available SR records (*n* = 1345) in the register to screen in Excel in December 2013. 51 records were excluded for having the status “Withdrawn” (*n* = 45) or for having no status given (*n* = 6); 294 records were excluded for not having performed at least one meta-analysis; 234 records were excluded for not having an effect size given for the first outcome; and 217 records were excluded for being incomplete. We organized the remaining 549 SR records by the Cochrane Review Groups (CRGs) responsible for their production. SRs were collected from three CRGs: Acute Respiratory Infections (ARI), Infectious Diseases (ID), and Development, Psychosocial and Learning Problems (DPLP). These three CRGs were selected by the research team as they contained the most SRs for analysis compared to other CRGs (ARI = 57; ID = 38; DPLP = 34), and represented three distinct clinical topic areas. All SRs from these three review groups were included in our final sample if they contained one or more meta-analysis (both continuous and/or dichotomous outcomes were eligible).

### Data extraction

For each SR, all of the studies included in the primary meta-analysis were listed, forming our reference standard. The primary meta-analysis was either for the identified primary outcome, or the meta-analysis presented first in the review, if the primary outcome was not specified. For each study included in each meta-analysis, we checked the authors’ list of references to studies included in the review and categorized them as published or unpublished (i.e., authors indicated “published data only” vs. “published and unpublished data” or “unpublished data only”). We examined the citations and associated database records to identify any non-English studies. We made particular effort to identify any non-English studies when searching the databases examined for our companion methods paper [23]. As it was possible that titles had been translated into English for the reviews’ reference lists, we checked full-text whenever the title of a study database record appeared in square brackets or when the source title was not English. Further, we identified any included dissertations by examining the citations (dissertations typically included “PhD” in the citation and place of publication was a university or academic institution). Finally, we examined the section of the reviews describing the searching methods and documented if the review authors specifically indicated that they searched for unpublished studies (or grey literature), dissertations, or non-English studies.

### Data analysis

For each review group and overall, we calculated: 1) the number of reviews that indicated they searched for each study type and the percentage relative to the total number of reviews; 2) the number of reviews that included each study type and the percentage relative to the total number that searched for the study type; and, 3) the number of each study type included and the percentage relative to the total number of studies included across the reviews.

For each meta-analysis that included at least one study representing one of our study types of interest, we re-analyzed the data excluding each study type. We used the same methods as the original meta-analysis (i.e., same summary measure and model). We calculated the percent of studies lost to each meta-analysis due to removal of each study type. We calculated the percent change in the point estimate and confidence interval width between the original and revised analysis. For point estimate, we ignored the direction of effect and considered only the magnitude. We categorized the change in point estimate as negligible (<5% change), small (5–10% change), moderate (11–20% change), large (more than 20%), and substantial (i.e., it was not possible to calculate an effect estimate as all the studies were categorized as a given study type, e.g., all were non-English). We also examined whether or not the result changed in statistical significance.

Data were managed using Excel Version 14.4.8 (Microsoft Corporation, Redmond, WA USA). Statistical analyses were conducted using RevMan 5.3 and SAS 9.4 (SAS institute Inc., Cary, NC USA).

## Results

Our analyses are based on 129 meta-analyses: 57 from ARI, 38 from ID, and 34 from DPLP. Citations and a description of the SRs used in our analysis are included in the Additional file 1. The median year of publication was 2007 (IQR 1996–2013). The median number of studies and participants were 3 (IQR 1–35) and 636.5 (IQR 30–4,400,266).

Table 1 shows the number of SRs that searched for and included non-English studies, unpublished studies, and dissertations. Table 1 also shows the number of each type of study that was included and the percentage relative to the total number of studies.

**Table 1:** Number of systematic reviews that searched for and included non-English studies, unpublished studies, and dissertations

Review Group	Total number of SRs	Number that searched for study type^a^ N (%)	Number that included study type^a^ N (%)	Total number of studies included in SR	Total number of study type^a^ included N (%)
Non-English Studies					
ARI	57	57 (100)	11 (19.3)	398	28 (7.0)
ID	38	38 (100)	4 (10.5)	238	6 (0.8)
DPLP	34	33 (97.1)	0 (0.0)	144	0 (0.0)
Total	129	128 (99.2)	15 (11.7)	780	34 (4.4)
Unpublished studies					
ARI	57	55 (96.5)	5 (7.3)	398	11 (2.8)
ID	38	38 (100)	2 (5.3)	238	3 (1.3)
DPLP	34	31 (91.2)	1 (3.2)	144	1 (0.7)
Total	129	124 (96.1)	8 (5.6)	780	15 (1.9)
Dissertations					
ARI	57	5 (8.8)	1 (20.0)	398	2 (0.5)
ID	38	1 (2.6)	1 (100)	238	2 (0.8)
DPLP	34	22 (64.7)	7 (31.8)	144	11 (7.6)
Total	129	28 (21.7)	9 (32.1)	780	15 (1.9)

*ARI* acute respiratory infections, *ID* infectious diseases, *DPLP* developmental, psychosocial and learning problems, *SR* systematic review

^a^study type refers to Non-English, unpublished, or dissertation

The majority of reviews across review groups searched for non-English studies; however, only 12% of reviews included non-English studies and these represented only 4% of the total studies contained across all reviews (*n* = 34/780). (See Additional file 1 for complete list of systematic reviews that included non-English language studies in their analyses). Among reviews that searched for non-English studies, there was a substantial difference across topic areas in terms of actually including non-English studies, with almost 20% of reviews in ARI compared to 0 in DPLP. Further, non-English studies represented 7% of all included studies in ARI reviews compared to 0 and 0.8% in DPLP and ID, respectively. The non-English studies included in ARI reviews were in the following languages: 32% French, 21% Spanish, 7% Chinese, 18% Italian, 14% German, 4% Turkish, and 4% Swedish. In ID reviews that included non-English studies, 50% of studies were Spanish, 33% were French, and 17% were Chinese.

Table 2 shows the results of the 15 meta-analyses where non-English studies were included, and the results when the non-English studies were removed. In nine cases the change in the effect estimate was negligible or small, and in two cases the change was moderate but no change in statistical significance. In two cases the change in effect estimate was large. There was a change in statistical significance in only one of these cases: the lower confidence interval changed from significant (0.01) to not significant (0.00); however, the conclusions of the review would not have changed as the authors concluded no clinically important difference between the two interventions. In two cases, all included studies were non-English; therefore, no effect estimate was available without the non-English studies. The topics of these reviews were acetylcysteine and carbocysteine for acute upper and lower respiratory tract infections in pediatric patients without chronic bronchopulmonary disease, and Chinese medicinal herbs for influenza. Change in confidence interval tended to be similar to change in point estimate with two exceptions. One study had a relatively small CI change despite a large point estimate difference, while another showed the opposite.

**Table 2:** Results of meta-analyses with and without Non-English studies

	Number of studies			Number of participants			Pooled estimate (95% CI) Summary measure		% change in effect size/CI width	Difference in estimates
Review	With non-English	Without non-English	% lost	With non-English	Without non-English	% lost	With non-English	Without non-English		
Acute respiratory infections										
Vitamin C for preventing and treating the common cold (CD000980)	29	26	10.3	11306	10899	3.6	0.95 (0.92, 0.98) RR	0.96 (0.93, 0.99)	1.1/0.0	Negligible
Epinephrine for bronchiolitis (CD003123)	6	5	16.7	995	965	3.0	0.67 (0.50, 0.89) RR	0.68 (0.51, 0.91)	1.5/2.6	Negligible
Procalcitonin to initiate or discontinue antibiotics in ARI (CD007498)	14	13	7.1	4211	4168	1.0	0.91 (0.70, 1.19) OR	0.90 (0.69, 1.19)	1.1/2.0	Negligible
Immunostimulants for preventing ARI in children (CD004974)	35	23	34.3	4060	2866	29.4	−1.24 (−1.54, -0.94) MD	−1.27 (−1.57, −0.96)	2.4/1.7	Negligible
Bronchodilators for bronchiolitis (CD001266)	24	23	4.2	1182	1149	2.8	−0.45 (−0.96, 0.05) MD	−0.41 (−0.97, 0.14)	8.9/9.9	Small
Glucocorticoids for croup (CD001955)	14	13	7.1	1031	998	3.2	−0.59 (−0.83, -0.35) SMD	−0.65 (−0.89, −0.41)	10.1/0.0	Small
Azithromycin for acute lower respiratory tract infections (CD001954)	15	14	6.7	2496	2446	2.0	1.09 (0.64, 1.85) RR	1.03 (0.60, 1.78)	5.5/2.5	Small
Systemic corticosteroids for acute sinusitis (CD008115)	4	2	50.0	869	459	47.2	1.40 (1.08, 1.81) RR	1.18 (1.05, 1.34)	15.7/60.3	Moderate
Antibiotics for the common cold and acute purulent rhinitis (CD000247)	6	5	16.7	1047	979	6.5	0.95 (0.59, 1.51) RR	1.11 (0.70, 1.76)	16.8/15.2	Moderate
Acetylcysteine and carbocysteine for acute upper and lower RTIs in paediatric patients without chronic broncho-pulmonary disease (CD003124)	3	0	100	139	0	100	−0.05 (−0.12, 0.02) RD	NA	NA	Substantial
Chinese medicinal herbs for influenza (CD004559)	2	0	100	197	0	100	1.32 (0.87, 2.00) RR	NA	NA	Substantial
Infectious diseases										
Vaccines for preventing malaria (CD006198)	3	2	33.3	307	237	22.8	1.05 (0.82, 1.35) RR	1.05 (0.82, 1.35)	0/0	Negligible
Probiotics for treating acute infectious diarrhoea (CD003048)	35	34	2.9	4555	4414	3.1	−24.76 (−33.61, −15.91) MD	−25.53 (−34.58, −16.49)	3.1/1.1	Negligible
High first dose quinine regimen for treating severe malaria (CD003341)	3	2	33.3	144	72	50.0	0.62 (0.19, 2.04) RR	0.43 (0.09, 2.05)	30.7/5.9	Large
Oral versus intravenous rehydration for treating dehydration due to gastroenteritis in children (CD004390)	18	15	16.7	1811	1436	20.7	0.04 (0.01, 0.07) RD	0.02 (0.00, 0.04)	50.0/33.3	Large (with change in statistical significance)

The majority of reviews searched for unpublished studies with some variation across groups (i.e., 91% for DPLP vs. 100% for ID). Only a very small percentage of reviews included unpublished studies (*n* = 8/124; 5.6%); likewise, these reflected a very small percentage of the total studies (*n* = 15/780; 1.9%). There was little variation across topics with very small numbers of unpublished studies included in each.

Table 3 shows the results of the eight meta-analyses that included unpublished studies. In four cases the change in effect estimates was negligible. In three cases the change was large, but none of the results changed in statistical significance. One study showed a small change in CI width, despite a large change in point estimate; otherwise changes in CI tended to be similar to changes in point estimate. One review included only unpublished studies; therefore, no effect estimate was available without the unpublished studies. The topic of the review was neuroaminidase inhibitors for preventing and treating influenza, a particularly high profile topic that relied heavily on industry reports and other regulatory documents [24].

**Table 3:** Results of meta-analyses with and without unpublished studies

	Number of studies			Number of participants			Pooled estimate (95% CI) Summary measure		% change in effect size/CI width	Difference in estimates
Review	With unpublished	Without unpublished	% lost	With unpublished	Without unpublished	% lost	With unpublished	Without unpublished		
Acute respiratory infections										
Corticosteroids for acute bacterial meningitis (CD004405)	25	24	4.0	4121	4036	2.1	0.90 (0.80, 1.01) RR	0.90 (0.80, 1.01)	0/0	Negligible
Vitamin C for preventing and treating the common cold (CD000980)	29	27	6.9	11306	11244	0.5	0.95 (0.92, 0.98) RR	0.95 (0.93, 0.98)	0/16.7	Negligible
Neuraminidase inhibitors for preventing and treating influenza in children (CD002744)	2	1	50.0	585	346	40.9	−0.13 (−0.21, −0.05) RD	−0.16 (−0.26, −0.05)	23.1/31.3	Large
Bronchodilators for bronchiolitis (CD001266)	24	22	8.3	1182	1113	5.8	−0.45 (−0.96, 0.05) MD	−0.29 (−0.79, 0.21)	35.6/1.0	Large
Neuraminidase inhibitors for preventing and treating influenza (CD008965)	5	0	100	3713	0	100	−21.29 (−29.59, −12.98) MD	NA	NA	-
Infectious diseases										
Artesunate versus quinine for treating severe malaria (CD005967)	8	6	25.0	7429	7198	3.1	0.71 (0.62, 0.80) RR	0.72 (0.64, 0.82)	1.4/0.0	Negligible
Probiotics for treating acute infectious diarrhoea (CD003048)	35	34	2.9	4555	4461	2.1	−24.76 (−33.61, −15.91) MD	−23.91 (−32.78, −15.03)	3.4/3.5	Negligible
Developmental, psychosocial and learning problems										
Iron therapy for improving psychomotor development and cognitive function in children under the age of three with iron deficiency anaemia (CD001444)	5	4	20.0	162	128	21.0	−1.25 (−4.56, 2.06) MD	−1.96 (−5.17, 3.15)	56.8/25.7	Large

There was wide variation across topics in searching for dissertations from 64.7% of reviews in DPLP compared to 2.6% in ID. Among the reviews that searched for dissertations, there was wide variation in the percentage that included dissertations from 20% in ARI to 100% in ID (although the latter was based on only one review that searched for dissertations). Overall, dissertations represented a very small percentage of included studies (*n* = 15/780; 1.9%), with variability across topics: for ARI and ID, dissertations represented less than 1% of included studies, while for DPLP they represented 7.6%.

Table 4 shows the results of the nine meta-analyses that included dissertations, and the results when the dissertations were removed. In all but one case the change in effect estimates was negligible or small, and changes in CI width tended to be similar to changes in magnitude to changes in point estimate. In one case the change in effect estimate was large and the statistical significance changed: the result with the dissertations was statistically significant (SMD −0.24, 95% CI −0.35, −0.13; 10 studies) and without dissertations the upper confidence interval rested on the null (SMD −0.19, 95% CI −0.38, 0.00; 5 studies). In both cases (with and without dissertations), the effect estimate was small in magnitude but was smaller (more conservative) without the dissertations. The topic of this review was kinship care for the safety, permanency, and well-being of children removed from the home for maltreatment.

**Table 4:** Results of meta-analyses with and without dissertations

	Number of studies			Number of participants			Pooled estimate (95% CI) Summary measure		% change in effect size/CI width	Difference in estimates
Review	With dissertations	Without dissertations	% lost	With dissertations	Without dissertations	% lost	With dissertations	Without dissertations		
Acute respiratory infections										
Vitamin C for preventing and treating the common cold (CD000980)	29	27	6.9	11306	11244	0.5	0.95 (0.92, 0.98) RR	0.95 (0.93, 0.98)	0/16.7	Negligible
Infectious diseases										
Oral iron supplements for children in malaria-endemic areas (CD006589)	13	11	15.4	NR	NR	NA	0.99 (0.90, 1.09) RR	0.95 (0.86, 1.05)	4.0/0.0	Negligible
Developmental, psychosocial and learning problems										
Household interventions for preventing domestic lead exposure in children (CD006047)	5	4	20.0	815	765	6.1	0.02 (−0.09, 0.12) MD	0.00 (−0.11, 0.11)	-/4.8	-
Phonics training for English-speaking poor readers (CD009115)	10	9	10.0	683	665	2.6	0.47 (0.06, 0.88) SMD	0.50 (0.06, 0.94)	6.4/7.3	Small
Music therapy for people with autism spectrum disorder (CD004381)	2	1	50.0	NR	NR	NA	0.50 (0.22, 0.79) SMD	0.48 (0.18, 0.77)	4.0/3.5	Negligible
Intermittent iron supplementation for improving nutrition and development in children 3.5under 12 years of age (CD009085)	10	9	10.0	1824	1774	2.7	0.51 (0.37, 0.72) RR	0.53 (0.38, 0.73)	3.9/0.0	Negligible
Cognitive-behavioral treatment for antisocial behavior in youth in residential treatment (CD005650)	4	3	25.0	560	417	25.5	0.87 (0.61, 1.25)	0.84 (0.55, 1.28)	3.5/14.1	Negligible
‘Scared Straight’ and other juvenile awareness programs for preventing juvenile delinquency (CD002796)	7	6	14.3	794	715	9.9	1.68 (1.20, 2.36)	1.71 (1.19, 2.46)	1.8/9.5	Negligible
Kinship care for the safety, permanency, and well-being of children removed from the home for maltreatment (CD006546)	10	5	50.0	2189	1396	36.2	−0.24 (−0.35, −0.13)	−0.19 (−0.38, 0.00)	20.8/72.7	Large (with change in statistical significance)

## Discussion

This study provides empiric evidence on the impact of searching for and including studies published in languages other than English, unpublished studies, and dissertations. The majority of SRs in our sample searched for non-English studies; however, these were included in a minority of reviews (12%) and represented less than 5% of all included studies. Moreover, there was a large or substantive change in results in only four reviews (among the total sample of 129). In two of these cases there were few included studies (2 and 3, respectively) and all were non-English. In two other cases the large change did not have an impact on the statistical or clinical significance of the findings. These results indicate that restricting the search and inclusion to English-only studies may not have an impact on the results of meta-analyses in the vast majority of cases. Searching for non-English studies should be considered on a case-by-case basis considering the topic area (due to the nature of the topic experts might expect evidence to be published or not in other languages, e.g., complementary and alternative medicine or diseases common in low-middle income countries) and volume of evidence (i.e., may be more necessary in areas where there is little evidence).

Likewise, the majority of SRs in our sample searched for unpublished studies but the vast majority did not include these (only 6% of reviews) and they represented only 2% of all included studies. In most cases the impact of including unpublished studies was small; only two of the eight meta-analyses including unpublished studies showed a large change in point estimates but in both cases there was no change in statistical significance of the result. In an additional case (of the eight meta-analyses), there were no published studies included; this review relied on unpublished industry reports and regulatory documents because of questions raised about the credibility of the published reports. The authors of this review had been questioned about the findings of an earlier version for which the conclusions were based on a pooled analysis, conducted by the manufacturer of the manufacturer-sponsored trials [24]. To address the concern, the authors set out to obtain the unpublished data from the drug manufacturer [24]. We might argue that this was a special case, and advise reviewers to seek unpublished data when the manufacturer has been heavily involved in a substantial proportion of published reports identified for inclusion. Further, we would advise that reviewers follow guidance on presenting information about vested interests (e.g., industry sponsored trials), and use this information when interpreting results and drawing conclusions [25].

Dissertations are a specific type of grey literature defined as a document supporting candidature for a doctorate degree that presents the candidate’s research and results [26]. One can assume that these documents undergo some extent of external review by content experts. We found variation across review groups with very few reviews in the ARI (9%) and ID (3%) groups searching for and including dissertations compared to 65% of DPLP reviews. Overall dissertations were included in only nine SRs (seven of these in DPLP, one each for ARI and ID) and represented less than 2% of all included studies. In the majority of cases the change in results was small or negligible. In one case, dissertations represented half of the included studies (five of ten) and there was a large change in the point estimate when dissertations were removed. Further, the result changed from statistically significant to not significant (lower confidence interval on the null). However, removing the dissertations resulted in a more conservative estimate, which may indicate that authors should carefully consider results when dissertations are included and the potential for overestimating treatment effects.

This study builds on work we recently published on the potential impact of prioritizing particular databases on the results of SRs, wherein we found that a limited number of databases provided the majority of relevant studies. Moreover, the results of meta-analyses based on studies contained in fewer databases did not differ, in the majority of cases, from the results of meta-analyses that included all identified studies [23]. However, we noted that the choice of database, and likewise decisions around searching for and including non-English studies, unpublished studies, and dissertations are likely topic dependent. Our results generally support reviewers to limit their searches in the interests of efficiencies without an important impact on results (in the vast majority of cases). Our results may be particularly relevant for rapid reviews, which are intended to produce evidence reports more quickly and efficiently than traditional SRs [27–29]. In rapid reviews, searching is one step that is typically modified to create efficiencies [29, 30]. Changes include searching fewer databases, limiting the search for grey literature, and restricting by language of publication (e.g., English only) [23]. Recent research found that end-users of SRs identified restrictions to searching as an acceptable trade-off in the interests of creating efficiencies in the review process [22].

Our study had several limitations; many of these were cited in our previous related publication [23]. First, we derived our sample from the CDSR; further, the SRs came from three review groups and focused on healthcare interventions and randomized controlled trials. Results may not be generalizable to all clinical areas, for non-traditional interventions, or for SRs of alternative research questions (e.g., diagnostic, prognostic) or study designs (e.g., observational or qualitative studies). Second, our sample included SRs that were already complete and we used as our reference standard the original search strategies and the included studies from completed Cochrane reviews. We cannot confirm the sensitivity of the original search strategies to retrieve all potentially relevant studies; however, Cochrane reviews are recognized as having gold standard methods (including criterion related to searching) and searching for unpublished studies and dissertations and inclusion of non-English studies are mandatory expectations of Cochrane reviews. Third, we used completed analyses of the primary outcomes from SRs. Focusing on the primary outcomes provided us with the most data from which to test our hypotheses; however, results from the SRs may have varied across outcomes. Fourth, to determine if included studies were non-English or dissertations, we examined reference lists from reviews, the studies’ database records and, when necessary, full-text manuscripts. Despite our efforts to accurately represent the contribution of non-English studies and dissertations, we may not have accounted for all instances of these study types due to inaccurate reference lists in original reviews and incorrect metadata in database records. Fifth, there are other sources of unpublished studies, such as clinical trials registries and conference proceedings that we did not specifically examine. Future research on these specific sources of study data will contribute empiric evidence to guide this important aspect of knowledge synthesis. Further, additional studies conducted prospectively and in different clinical areas would be valuable; our study provides data based on a small proportion of published Cochrane reviews (129 meta-analyses from approximately 7000 total reviews in the CDSR). Sixth, we used a pragmatic approach to classify the extent of change as negligible, small, moderate, large or substantial. This may be too simplistic from a clinical point of view where other factors may be considered such as the nature of the outcome and the extent of heterogeneity. An alternative approach to classifying the change would be to ask clinical experts about the clinical significance of the change; however this was beyond the scope of the present work. Finally it should be noted that when looking at differences in results between meta-analyses, we analyzed reviews which identified non-English and unpublished studies and dissertations. It is possible these types of studies did exist even in the reviews that were unable to find them—they were simply too difficult for the searching to locate. We are unable to know if there is an inherent difference between removing studies when they are found (what we did) and adding them when they could not be found. Future research is needed to investigate the impact on SRs of including data from sources that are typically unavailable to reviewers.

## Conclusions

This study provides quantitative data regarding the potential impact on meta-analysis results of excluding studies published in non-English languages, as well as unpublished studies and dissertations. We found that the vast majority of SRs searched for non-English and unpublished studies; however, these represented a very small proportion of included studies and rarely impacted the results and conclusions of the review. Inclusion of these study types may have an impact in situations where there are very few relevant studies, or where there are questionable vested interests identified in the published literature. We found substantial variation in whether SRs searched for dissertations; in the majority of reviews that included dissertations, these had little impact on the results and in fact may overestimate treatment effects. The findings from this study may be useful to optimize the conduct of SRs and guide the development of methods for rapid reviews. Future research in different clinical areas, and for other select types of grey literature, will help establish best practices for literature searching to support evidence syntheses.

## Additional file

Additional file 1: Description of Included Systematic Reviews. This file contains a detailed listing of the systematic reviews that were included in the analysis, as well as a summary of the reviews, by Cochrane group, which included non-English language studies, unpublished studies and/or dissertations. (DOCX 62 kb)

## References

[1] Lavis JN, Davies HT, Gruen RL, Walshe K, Farquhar CM (2006). Working within and beyond the Cochrane Collaboration to make systematic reviews more useful to healthcare managers and policy makers. Healthc Policy.

[2] Lefebvre C, Manheimer E, Glanville J, Higgins JP, Green S (2011). Chapter 6: Searching for studies. Cochrane Handbook for Systematic Reviews of Interventions Version 5.1.0. The Cochrane Collaboration.

[3] Sterne JA, Egger M, Moher D, Higgins JP, Green S (2011). Chapter 10: Addressing reporting biases. Cochrane Handbook for Systematic Reviews of Interventions Version 5.1.0. The Cochrane Collaboration.

[4] Higgins JPT, Lasserson T, Chandler J, Tovey D, Churchill R. Methodological Expectations of Cochrane Intervention Reviews (MECIR): standards for the conduct and reporting of new Cochrane Intervention Reviews, reporting of protocols and the planning, conduct and reporting of updates. Cochrane; 2016 [cited 2017 Jan 3]. Available from: http://methods.cochrane.org/sites/default/files/public/uploads/mecir_printed_booklet_final.pdf.

[5] Institute of Medicine (2011). Finding what works in health care: standards for systematic reviews.

[6] Balshem H, Stevens A, Ansari M, Norris S, Kansagara D, Shamliyan T, Chou R, Chung M, Moher D, Dickersin K (2013). Finding grey literature evidence and assessing for outcome and analysis reporting biases when comparing medical interventions: AHRQ and the Effective Health Care Program. Methods Guide for Comparative Effectiveness Reviews.

[7] Lefebvre C, Manheimer E, Glanville J, Higgins JP, Green S (2011). Chapter 6.2.3: Unpublished and ongoing studies. Cochrane Handbook for Systematic Reviews of Interventions Version 5.1.0. The Cochrane Collaboration.

[8] Egger M, Juni P, Bartlett C, Holenstein F, Sterne J. How important are comprehensive literature searches and the assessment of trial quality in systematic reviews?: Empirical study. Health Technol Asses. 2003;7(1). [cited 2017 Jan 4] Available from Medline: https://www.ncbi.nlm.nih.gov/pubmed/12583822. 12583822

[9] Jefferson T, Jones MA, Doshi P, Del Mar CB, Heneghan CJ, Hama R, Thompson MJ. Neuraminidase inhibitors for preventing and treating influenza in healthy adults and children. Cochrane Database of Syst Rev. 2012 [cited 2017 Jan 3];Jan 18. Available from Medline: https://www.ncbi.nlm.nih.gov/pubmed/22258996. 10.1002/14651858.CD008965.pub3 22258996

[10] Hart B, Lundh A, Bero L (2012). Effect of reporting bias on meta-analyses of drug trials: reanalysis of meta-analyses. BMJ.

[11] Turner EH, Knoepflmacher D, Shapley L (2012). Publication bias in antipsychotic trials: an analysis of efficacy comparing the published literature to the US Food and Drug Administration database. PLoS Med.

[12] Golder S, Loke YK, Wright K, Norman G (2016). Reporting of adverse events in published and unpublished studies of health care interventions: a systematic review. PLoS Med.

[13] Whittington CJ, Kendall T, Fonagy P, Cottrell D, Cotgrove A, Boddington E (2004). Selective serotonin reuptake inhibitors in childhood depression: systematic review of published versus unpublished data. Lancet.

[14] Lefebvre C, Manheimer E, Glanville J, Higgins JP, Green S (2011). Chapter 6.2.1.7 Dissertations and theses databases. Cochrane Handbook for Systematic Reviews of Interventions Version 5.1.0. The Cochrane Collaboration.

[15] Moher D, Pham B, Lawson ML, Klassen TP (2003). The inclusion of reports of randomised trials published in languages other than English in systematic reviews. Health Technol Assess.

[16] Egger M, Zellweger-Zähner T, Schneider M, Junker C, Lengeler C, Antes G (1997). Language bias in randomised controlled trials published in English and German. Lancet.

[17] Heres S, Wagenpfeil S, Hamann J, Kissling W, Leucht S (2004). Language bias in neuroscience—is the Tower of Babel located in Germany?. Eur Psychiatry.

[18] Vickers A, Goyal N, Harland R, Rees R (1998). Do certain countries produce only positive results? A systematic review of controlled trials. Control Clin Trials.

[19] Pham B, Klassen TP, Lawson ML, Moher D (2005). Language of publication restrictions in systematic reviews gave different results depending on whether the intervention was conventional or complementary. J Clin Epidemiol.

[20] Royle PL, Bain L, Waugh NR (2005). Sources of evidence for systematic reviews of interventions in diabetes. Diabet Med.

[21] Royle P, Waugh N. Literature searching for clinical and cost-effectiveness studies used in health technology assessment reports carried out for the National Institute for Clinical Excellence appraisal system. Health Technol Assess. 2003;7(34):iii, ix-x, 1–51. [cited 2016 Jun 7] Available from Medline: http://www.ncbi.nlm.nih.gov/pubmed/14609481 10.3310/hta7340 14609481

[22] Hartling L, Guise JM, Hempel S, Featherstone R, Mitchell MD, Motu’apuaka ML (2016). EPC Methods: AHRQ End-User Perspectives of Rapid Reviews.

[23] Hartling L, Featherstone R, Nuspl M, Shave K, Dryden DM, Vandermeer B (2016). The contribution of databases to the results of systematic reviews: a cross-sectional study. BMC Med Res Methodol.

[24] Jones M, Jefferson T, Doshi P, Del Mar C, Heneghan C, Onakpoya I (2015). Commentary on Cochrane review of neuraminidase inhibitors for preventing and treating influenza in healthy adults and children. Clin Microbiol Infect.

[25] Higgins JPT, Altman DG, Sterne JAC, Higgins JPT, Green S (2011). Chapter 8: Assessing risk of bias in included studies. Cochrane Handbook for Systematic Reviews of Interventions Version 5.1.0. The Cochrane Collaboration.

[26] Wikipedia contributors. Thesis. Wikipedia, The Free Encyclopedia. 2017, [cited 2017 Jan 3] Available from: https://en.wikipedia.org/w/index.php?title=Thesis&oldid=758058152.

[27] Ganann R, Ciliska D, Thomas H (2010). Expediting systematic reviews: methods and implications of rapid reviews. Implement Sci.

[28] Khangura S, Polisena J, Clifford TJ, Farrah K, Kamel C (2014). Rapid review: an emerging approach to evidence synthesis in health technology assessment. Int J Technol Assess Health Care.

[29] Hartling L, Guise J-M, Kato E, Anderson J, Belinson S, Berliner E (2015). A taxonomy of rapid reviews links report types and methods to specific decision-making contexts. J Clin Epidemiol.

[30] Tricco AC, Antony J, Zarin W, Strifler L, Ghassemi M, Ivory J (2015). A scoping review of rapid review methods. BMC Med.

