# Supplementary material for: Grey literature in systematic reviews: a cross-sectional study of the contribution of non-English reports, unpublished studies and dissertations to the results of meta-analyses in child-relevant reviews
Source: BMC Med Res Methodol. 2017 Apr 19;17:64. doi: 10.1186/s12874-017-0347-z (PMC5395863; doi:10.1186/s12874-017-0347-z)
Supplement: Additional file 1: — Description of Included Systematic Reviews. This file contains a detailed listing of the systematic reviews that were included in the analysis, as well as a summary of the reviews, by Cochrane group, which included non-English language studies, unpublished studies and/or dissertations. (DOCX 62 kb) [file 12874_2017_347_MOESM1_ESM.docx]

**Additional File**

**Description of included systematic reviews**

|  | Acute Respiratory Infections  range (median) | Infectious Diseases  range (median) | Developmental Psychosocial and Learning Problems  range (median) | Overall |
| --- | --- | --- | --- | --- |
| Year of initial publication | 1998-2013 (2007) | 1996-2013 (2004) | 1999-2013 (2009.5) | 1996-2013 (2007) |
| Year of publication used in this study | 2004-2013 (2012) | 1999-2014 (2008.5) | 2000-2013 (2011) | 1999-2014 (2011) |
| Number of included studies overall | 3-73 (10) | 2-71 (12) | 2-62 (8) | 2-73 (10) |
| Number of studies in primary/first meta-analysis | 1-35 (4) | 1-35 (3) | 1-17 (3) | 1-35 (3) |
| Number of participants in primary/first meta-analysis | 31-905,412 (1,031) | 43-4,400,266 (553) | 30-5,078 (308) | 30-4,400,266 (636.5) |

**57 systematic reviews with a least one meta-analysis from the Acute Respiratory Infections (ARI) Cochrane Review Group (notations indicate number of non-English language studies, unpublished studies and/or dissertations included in each review)**

1. AlBalawi Zaina H, Othman Sahar S, AlFaleh K. Intranasal ipratropium bromide for the common cold. Cochrane Database of Systematic Reviews [Internet]. 2013; (6). Available from: <http://onlinelibrary.wiley.com/doi/10.1002/14651858.CD008231.pub3> [non-English = 0 ; unpublished = 0 ; dissertations = 0]

2. Altamimi S, Khalil A, Khalaiwi Khalid A, Milner Ruth A, Pusic Martin V, Al Othman Mohammed A. Short-term late-generation antibiotics versus longer term penicillin for acute streptococcal pharyngitis in children. Cochrane Database of Systematic Reviews [Internet]. 2012; (8). Available from: <http://onlinelibrary.wiley.com/doi/10.1002/14651858.CD004872.pub3> [non-English = 0 ; unpublished = 0 ; dissertations = 0]

3. Alves Galvão Márcia G, Rocha Crispino Santos Marilene A, Alves da Cunha Antonio JL. Amantadine and rimantadine for influenza A in children and the elderly. Cochrane Database of Systematic Reviews [Internet]. 2012; (1). Available from: <http://onlinelibrary.wiley.com/doi/10.1002/14651858.CD002745.pub3> [non-English = 0 ; unpublished = 0 ; dissertations = 0]

4. Andabaka T, Nickerson Jason W, Rojas-Reyes Maria X, Rueda Juan D, Bacic Vrca V, Barsic B. Monoclonal antibody for reducing the risk of respiratory syncytial virus infection in children. Cochrane Database of Systematic Reviews [Internet]. 2013; (4). Available from: <http://onlinelibrary.wiley.com/doi/10.1002/14651858.CD006602.pub4> [non-English = 0 ; unpublished = 0 ; dissertations = 0]

5. Azarpazhooh A, Limeback H, Lawrence Herenia P, Shah Prakeshkumar S. Xylitol for preventing acute otitis media in children up to 12 years of age. Cochrane Database of Systematic Reviews [Internet]. 2011; (11). Available from: <http://onlinelibrary.wiley.com/doi/10.1002/14651858.CD007095.pub2> [non-English = 0 ; unpublished = 0 ; dissertations = 0]

6. Bar-On Edna S, Goldberg E, Hellmann S, Leibovici L. Combined DTP-HBV-HIB vaccine versus separately administered DTP-HBV and HIB vaccines for primary prevention of diphtheria, tetanus, pertussis, hepatitis B and Haemophilus influenzae B (HIB). Cochrane Database of Systematic Reviews [Internet]. 2012; (4). Available from: <http://onlinelibrary.wiley.com/doi/10.1002/14651858.CD005530.pub3> [non-English = 0 ; unpublished = 0 ; dissertations = 0]

7. Becker Lorne A, Hom J, Villasis-Keever M, van der Wouden Johannes C. Beta2-agonists for acute bronchitis. Cochrane Database of Systematic Reviews [Internet]. 2011; (7). Available from: <http://onlinelibrary.wiley.com/doi/10.1002/14651858.CD001726.pub4> [non-English = 0 ; unpublished = 0 ; dissertations = 0]

8. Bjerre Lise M, Verheij Theo JM, Kochen Michael M. Antibiotics for community acquired pneumonia in adult outpatients. Cochrane Database of Systematic Reviews [Internet]. 2009; (4). Available from: <http://onlinelibrary.wiley.com/doi/10.1002/14651858.CD002109.pub3> [non-English = 0 ; unpublished = 0 ; dissertations = 0]

9. Bjornson C, Russell K, Vandermeer B, Klassen Terry P, Johnson David W. Nebulized epinephrine for croup in children. Cochrane Database of Systematic Reviews [Internet]. 2013; (10). Available from: <http://onlinelibrary.wiley.com/doi/10.1002/14651858.CD006619.pub3> [non-English = 0 ; unpublished = 0 ; dissertations = 0]

10. Brouwer Matthijs C, McIntyre P, Prasad K, van de Beek D. Corticosteroids for acute bacterial meningitis. Cochrane Database of Systematic Reviews [Internet]. 2013; (6). Available from: <http://onlinelibrary.wiley.com/doi/10.1002/14651858.CD004405.pub4> [non-English = 0 ; unpublished = 1 ; dissertations = 0]

11. Chalumeau M, Duijvestijn Yvonne CM. Acetylcysteine and carbocysteine for acute upper and lower respiratory tract infections in paediatric patients without chronic broncho-pulmonary disease. Cochrane Database of Systematic Reviews [Internet]. 2013; (5). Available from: <http://onlinelibrary.wiley.com/doi/10.1002/14651858.CD003124.pub4> [non-English = 3 ; unpublished = 0 ; dissertations = 0]

12. Chang Christina C, Cheng Allen C, Chang Anne B. Over-the-counter (OTC) medications to reduce cough as an adjunct to antibiotics for acute pneumonia in children and adults. Cochrane Database of Systematic Reviews [Internet]. 2012; (2). Available from: <http://onlinelibrary.wiley.com/doi/10.1002/14651858.CD006088.pub3> [non-English = 0 ; unpublished = 0 ; dissertations = 0]

13. Chen Y, Li K, Pu H, Wu T. Corticosteroids for pneumonia. Cochrane Database of Systematic Reviews [Internet]. 2011; (3). Available from: <http://onlinelibrary.wiley.com/doi/10.1002/14651858.CD007720.pub2> [non-English = 0 ; unpublished = 0 ; dissertations = 0]

14. De Sutter An IM, van Driel Mieke L, Kumar Anna A, Lesslar O, Skrt A. Oral antihistamine-decongestant-analgesic combinations for the common cold. Cochrane Database of Systematic Reviews [Internet]. 2012; (2). Available from: <http://onlinelibrary.wiley.com/doi/10.1002/14651858.CD004976.pub3> [non-English = 0 ; unpublished = 0 ; dissertations = 0]

15. Del-Rio-Navarro Blanca E, Espinosa-Rosales Francisco J, Flenady V, Sienra-Monge Juan JL. Immunostimulants for preventing respiratory tract infection in children. Cochrane Database of Systematic Reviews [Internet]. 2006; (4). Available from: <http://onlinelibrary.wiley.com/doi/10.1002/14651858.CD004974.pub2> [non-English = 12 ; unpublished = 0 ; dissertations = 0]

16. Doan Q, Enarson P, Kissoon N, Klassen Terry P, Johnson David W. Rapid viral diagnosis for acute febrile respiratory illness in children in the Emergency Department. Cochrane Database of Systematic Reviews [Internet]. 2012; (5). Available from: <http://onlinelibrary.wiley.com/doi/10.1002/14651858.CD006452.pub3> [non-English = 0 ; unpublished = 0 ; dissertations = 0]

17. Ekeland E, Heian F, Hagen Kåre B, Abbott Jo M, Nordheim L. Exercise to improve self-esteem in children and young people. Cochrane Database of Systematic Reviews [Internet]. 2004; (1). Available from: <http://onlinelibrary.wiley.com/doi/10.1002/14651858.CD003683.pub2> [non-English = 0 ; unpublished = 0 ; dissertations = 0]

18. Enriquez A, Chu IW, Mellis C, Lin W-Y. Nebulised deoxyribonuclease for viral bronchiolitis in children younger than 24 months. Cochrane Database of Systematic Reviews [Internet]. 2012; (11). Available from: <http://onlinelibrary.wiley.com/doi/10.1002/14651858.CD008395.pub2> [non-English = 0 ; unpublished = 0 ; dissertations = 0]

19. Gadomski Anne M, Brower M. Bronchodilators for bronchiolitis. Cochrane Database of Systematic Reviews [Internet]. 2010; (12). Available from: <http://onlinelibrary.wiley.com/doi/10.1002/14651858.CD001266.pub3> [non-English = 1 ; unpublished = 2 ; dissertations = 0]

20. Gulani A, Sachdev Harshpal S. Zinc supplements for preventing otitis media. Cochrane Database of Systematic Reviews [Internet]. 2012; (4). Available from: <http://onlinelibrary.wiley.com/doi/10.1002/14651858.CD006639.pub3> [non-English = 0 ; unpublished = 0 ; dissertations = 0]

21. Haider Batool A, Lassi Zohra S, Ahmed A, Bhutta Zulfiqar A. Zinc supplementation as an adjunct to antibiotics in the treatment of pneumonia in children 2 to 59 months of age. Cochrane Database of Systematic Reviews [Internet]. 2011; (10). Available from: <http://onlinelibrary.wiley.com/doi/10.1002/14651858.CD007368.pub2> [non-English = 0 ; unpublished = 0 ; dissertations = 0]

22. Hartling L, Bialy Liza M, Vandermeer B, Tjosvold L, Johnson David W, Plint Amy C, et al. Epinephrine for bronchiolitis. Cochrane Database of Systematic Reviews [Internet]. 2011; (6). Available from: <http://onlinelibrary.wiley.com/doi/10.1002/14651858.CD003123.pub3> [non-English = 1 ; unpublished = 0 ; dissertations = 0]

23. Hayward G, Thompson Matthew J, Perera R, Glasziou Paul P, Del Mar Chris B, Heneghan Carl J. Corticosteroids as standalone or add-on treatment for sore throat. Cochrane Database of Systematic Reviews [Internet]. 2012; (10). Available from: <http://onlinelibrary.wiley.com/doi/10.1002/14651858.CD008268.pub2> [non-English = 0 ; unpublished = 0 ; dissertations = 0]

24. Hemilä H, Chalker E. Vitamin C for preventing and treating the common cold. Cochrane Database of Systematic Reviews [Internet]. 2013; (1). Available from: <http://onlinelibrary.wiley.com/doi/10.1002/14651858.CD000980.pub4> [non-English = 3 ; unpublished = 2 ; dissertations = 2]

25. Jefferson T, Del Mar Chris B, Dooley L, Ferroni E, Al-Ansary Lubna A, Bawazeer Ghada A, et al. Physical interventions to interrupt or reduce the spread of respiratory viruses. Cochrane Database of Systematic Reviews [Internet]. 2011; (7). Available from: <http://onlinelibrary.wiley.com/doi/10.1002/14651858.CD006207.pub4> [non-English = 0 ; unpublished = 0 ; dissertations = 0]

26. Jefferson T, Jones Mark A, Doshi P, Del Mar Chris B, Heneghan Carl J, Hama R, et al. Neuraminidase inhibitors for preventing and treating influenza in healthy adults and children. Cochrane Database of Systematic Reviews [Internet]. 2012; (1). Available from: <http://onlinelibrary.wiley.com/doi/10.1002/14651858.CD008965.pub3> [non-English = 0 ; unpublished = 5 ; dissertations = 0]

27. Jefferson T, Rivetti A, Di Pietrantonj C, Demicheli V, Ferroni E. Vaccines for preventing influenza in healthy children. Cochrane Database of Systematic Reviews [Internet]. 2012; (8). Available from: <http://onlinelibrary.wiley.com/doi/10.1002/14651858.CD004879.pub4> [non-English = 0 ; unpublished = 0 ; dissertations = 0]

28. Jiang L, Deng L, Wu T. Chinese medicinal herbs for influenza. Cochrane Database of Systematic Reviews [Internet]. 2013; (3). Available from: <http://onlinelibrary.wiley.com/doi/10.1002/14651858.CD004559.pub4> [non-English = 2 ; unpublished = 0 ; dissertations = 0]

29. Kabra Sushil K, Lodha R. Antibiotics for preventing complications in children with measles. Cochrane Database of Systematic Reviews [Internet]. 2013; (8). Available from: <http://onlinelibrary.wiley.com/doi/10.1002/14651858.CD001477.pub4> [non-English = 0 ; unpublished = 0 ; dissertations = 0]

30. Kassel Jessica C, King D, Spurling Geoffrey KP. Saline nasal irrigation for acute upper respiratory tract infections. Cochrane Database of Systematic Reviews [Internet]. 2010; (3). Available from: <http://onlinelibrary.wiley.com/doi/10.1002/14651858.CD006821.pub2> [non-English = 0 ; unpublished = 0 ; dissertations = 0]

31. Kenealy T, Arroll B. Antibiotics for the common cold and acute purulent rhinitis. Cochrane Database of Systematic Reviews [Internet]. 2013; (6). Available from: <http://onlinelibrary.wiley.com/doi/10.1002/14651858.CD000247.pub3> [non-English = 1 ; unpublished = 0 ; dissertations = 0]

32. Kim Soo Y, Chang Y-J, Cho Hye M, Hwang Y-W, Moon Yoo S. Non-steroidal anti-inflammatory drugs for the common cold. Cochrane Database of Systematic Reviews [Internet]. 2013; (6). Available from: <http://onlinelibrary.wiley.com/doi/10.1002/14651858.CD006362.pub3> [non-English = 0 ; unpublished = 0 ; dissertations = 0]

33. Klassen Terry P, Hartling L. Acyclovir for treating varicella in otherwise healthy children and adolescents. Cochrane Database of Systematic Reviews [Internet]. 2005; (4). Available from: <http://onlinelibrary.wiley.com/doi/10.1002/14651858.CD002980.pub3> [non-English = 0 ; unpublished = 0 ; dissertations = 0]

34. Kozyrskyj Anita L, Klassen Terry P, Moffatt M, Harvey K. Short-course antibiotics for acute otitis media. Cochrane Database of Systematic Reviews [Internet]. 2010; (9). Available from: <http://onlinelibrary.wiley.com/doi/10.1002/14651858.CD001095.pub2> [non-English = 0 ; unpublished = 0 ; dissertations = 0]

35. Laopaiboon M, Panpanich R, Lerttrakarnnon P. Azithromycin for acute lower respiratory tract infections. Cochrane Database of Systematic Reviews [Internet]. 2008; (1). Available from: <http://onlinelibrary.wiley.com/doi/10.1002/14651858.CD001954.pub3> [non-English = 1 ; unpublished = 0 ; dissertations = 0]

36. Lassi Zohra S, Haider Batool A, Bhutta Zulfiqar A. Zinc supplementation for the prevention of pneumonia in children aged 2 months to 59 months. Cochrane Database of Systematic Reviews [Internet]. 2010; (12). Available from: <http://onlinelibrary.wiley.com/doi/10.1002/14651858.CD005978.pub2> [non-English = 0 ; unpublished = 0 ; dissertations = 0]

37. Leach Amanda J, Morris Peter S. Antibiotics for the prevention of acute and chronic suppurative otitis media in children. Cochrane Database of Systematic Reviews [Internet]. 2006; (4). Available from: <http://onlinelibrary.wiley.com/doi/10.1002/14651858.CD004401.pub2> [non-English = 0 ; unpublished = 0 ; dissertations = 0]

38. Liet J-M, Ducruet T, Gupta V, Cambonie G. Heliox inhalation therapy for bronchiolitis in infants. Cochrane Database of Systematic Reviews [Internet]. 2010; (4). Available from: <http://onlinelibrary.wiley.com/doi/10.1002/14651858.CD006915.pub2> [non-English = 0 ; unpublished = 0 ; dissertations = 0]

39. Lucero Marilla G, Dulalia Vernoni E, Nillos Leilani T, Williams G, Parreño Rhea Angela N, Nohynek H, et al. Pneumococcal conjugate vaccines for preventing vaccine-type invasive pneumococcal disease and X-ray defined pneumonia in children less than two years of age. Cochrane Database of Systematic Reviews [Internet]. 2009; (4). Available from: <http://onlinelibrary.wiley.com/doi/10.1002/14651858.CD004977.pub2> [non-English = 0 ; unpublished = 0 ; dissertations = 0]

40. Mathie Robert T, Frye J, Fisher P. Homeopathic Oscillococcinum® for preventing and treating influenza and influenza-like illness. Cochrane Database of Systematic Reviews [Internet]. 2012; (12). Available from: <http://onlinelibrary.wiley.com/doi/10.1002/14651858.CD001957.pub5> [non-English = 0 ; unpublished = 0 ; dissertations = 0]

41. Patel M, Lee C-k. Polysaccharide vaccines for preventing serogroup A meningococcal meningitis. Cochrane Database of Systematic Reviews [Internet]. 2005; (1). Available from: <http://onlinelibrary.wiley.com/doi/10.1002/14651858.CD001093.pub2> [non-English = 0 ; unpublished = 0 ; dissertations = 0]

42. Prasad K, Kumar A, Singhal T, Gupta Praveen K. Third generation cephalosporins versus conventional antibiotics for treating acute bacterial meningitis. Cochrane Database of Systematic Reviews [Internet]. 2007; (4). Available from: <http://onlinelibrary.wiley.com/doi/10.1002/14651858.CD001832.pub3> [non-English = 0 ; unpublished = 0 ; dissertations = 0]

43. Ratilal Bernardo O, Costa J, Sampaio C, Pappamikail L. Antibiotic prophylaxis for preventing meningitis in patients with basilar skull fractures. Cochrane Database of Systematic Reviews [Internet]. 2011; (8). Available from: <http://onlinelibrary.wiley.com/doi/10.1002/14651858.CD004884.pub3> [non-English = 0 ; unpublished = 0 ; dissertations = 0]

44. Rojas-Reyes Maria X, Granados Rugeles C, Charry-Anzola Laura P. Oxygen therapy for lower respiratory tract infections in children between 3 months and 15 years of age. Cochrane Database of Systematic Reviews [Internet]. 2009; (1). Available from: <http://onlinelibrary.wiley.com/doi/10.1002/14651858.CD005975.pub2> [non-English = 0 ; unpublished = 0 ; dissertations = 0]

45. Russell Kelly F, Liang Y, O'Gorman K, Johnson David W, Klassen Terry P. Glucocorticoids for croup. Cochrane Database of Systematic Reviews [Internet]. 2011; (1). Available from: <http://onlinelibrary.wiley.com/doi/10.1002/14651858.CD001955.pub3> [non-English = 1 ; unpublished = 0 ; dissertations = 0]

46. Schuetz P, Müller B, Christ-Crain M, Stolz D, Tamm M, Bouadma L, et al. Procalcitonin to initiate or discontinue antibiotics in acute respiratory tract infections. Cochrane Database of Systematic Reviews [Internet]. 2012; (9). Available from: <http://onlinelibrary.wiley.com/doi/10.1002/14651858.CD007498.pub2> [non-English = 1 ; unpublished = 0 ; dissertations = 0]

47. Singh M, Das Rashmi R. Zinc for the common cold. Cochrane Database of Systematic Reviews [Internet]. 2013; (6). Available from: <http://onlinelibrary.wiley.com/doi/10.1002/14651858.CD001364.pub4> [non-English = 0 ; unpublished = 0 ; dissertations = 0]

48. Singh M, Singh M. Heated, humidified air for the common cold. Cochrane Database of Systematic Reviews [Internet]. 2013; (6). Available from: <http://onlinelibrary.wiley.com/doi/10.1002/14651858.CD001728.pub5> [non-English = 0 ; unpublished = 0 ; dissertations = 0]

49. Spurling Geoffrey KP, Doust J, Del Mar Chris B, Eriksson L. Antibiotics for bronchiolitis in children. Cochrane Database of Systematic Reviews [Internet]. 2011; (6). Available from: <http://onlinelibrary.wiley.com/doi/10.1002/14651858.CD005189.pub3> [non-English = 0 ; unpublished = 0 ; dissertations = 0]

50. van Driel Mieke L, De Sutter An IM, Keber N, Habraken H, Christiaens T. Different antibiotic treatments for group A streptococcal pharyngitis. Cochrane Database of Systematic Reviews [Internet]. 2013; (4). Available from: <http://onlinelibrary.wiley.com/doi/10.1002/14651858.CD004406.pub3> [non-English = 0 ; unpublished = 0 ; dissertations = 0]

51. Venekamp Roderick P, Thompson Matthew J, Hayward G, Heneghan Carl J, Del Mar Chris B, Perera R, et al. Systemic corticosteroids for acute sinusitis. Cochrane Database of Systematic Reviews [Internet]. 2011; (12). Available from: <http://onlinelibrary.wiley.com/doi/10.1002/14651858.CD008115.pub2> [non-English = 2 ; unpublished = 0 ; dissertations = 0]

52. Wall Emma CB, Ajdukiewicz Katherine MB, Heyderman Robert S, Garner P. Osmotic therapies added to antibiotics for acute bacterial meningitis. Cochrane Database of Systematic Reviews [Internet]. 2013; (3). Available from: <http://onlinelibrary.wiley.com/doi/10.1002/14651858.CD008806.pub2> [non-English = 0 ; unpublished = 0 ; dissertations = 0]

53. Wang K, Shun-Shin M, Gill P, Perera R, Harnden A. Neuraminidase inhibitors for preventing and treating influenza in children (published trials only). Cochrane Database of Systematic Reviews [Internet]. 2012; (4). Available from: <http://onlinelibrary.wiley.com/doi/10.1002/14651858.CD002744.pub4> [non-English = 0 ; unpublished = 1 ; dissertations = 0]

54. Wu T, Ni J, Wei J. Vitamin A for non-measles pneumonia in children. Cochrane Database of Systematic Reviews [Internet]. 2005; (3). Available from: <http://onlinelibrary.wiley.com/doi/10.1002/14651858.CD003700.pub2> [non-English = 0 ; unpublished = 0 ; dissertations = 0]

55. Zalmanovici Trestioreanu A, Yaphe J. Intranasal steroids for acute sinusitis. Cochrane Database of Systematic Reviews [Internet]. 2013; (12). Available from: <http://onlinelibrary.wiley.com/doi/10.1002/14651858.CD005149.pub4> [non-English = 0 ; unpublished = 0 ; dissertations = 0]

56. Zhang L, Mendoza-Sassi Raúl A, Wainwright C, Klassen Terry P. Nebulised hypertonic saline solution for acute bronchiolitis in infants. Cochrane Database of Systematic Reviews [Internet]. 2013; (7). Available from: <http://onlinelibrary.wiley.com/doi/10.1002/14651858.CD006458.pub3> [non-English = 0 ; unpublished = 0 ; dissertations = 0]

57. Zhang L, Prietsch Sílvio OM, Axelsson I, Halperin Scott A. Acellular vaccines for preventing whooping cough in children. Cochrane Database of Systematic Reviews [Internet]. 2012; (3). Available from: <http://onlinelibrary.wiley.com/doi/10.1002/14651858.CD001478.pub5> [non-English = 0 ; unpublished = 0 ; dissertations = 0]

**38 systematic reviews with a least one meta-analysis from the Infectious Diseases (ID) Cochrane Review Group**

1. Afolabi Bosede B, Okoromah Christy AN. Intramuscular arteether for treating severe malaria. Cochrane Database of Systematic Reviews [Internet]. 2004; (4). Available from: <http://onlinelibrary.wiley.com/doi/10.1002/14651858.CD004391.pub2> [non-English = 0 ; unpublished = 0 ; dissertations = 0]

2. Allen Stephen J, Martinez Elizabeth G, Gregorio Germana V, Dans Leonila F. Probiotics for treating acute infectious diarrhoea. Cochrane Database of Systematic Reviews [Internet]. 2010; (11). Available from: <http://onlinelibrary.wiley.com/doi/10.1002/14651858.CD003048.pub3> [non-English = 1 ; unpublished = 1 ; dissertations = 0]

3. Anwar E, Goldberg E, Fraser A, Acosta Camilo J, Paul M, Leibovici L. Vaccines for preventing typhoid fever. Cochrane Database of Systematic Reviews [Internet]. 2014; (1). Available from: <http://onlinelibrary.wiley.com/doi/10.1002/14651858.CD001261.pub3> [non-English = 0 ; unpublished = 0 ; dissertations = 0]

4. Bernaola Aponte G, Bada Mancilla Carlos A, Carreazo Nilton Y, Rojas Galarza Raúl A. Probiotics for treating persistent diarrhoea in children. Cochrane Database of Systematic Reviews [Internet]. 2013; (8). Available from: <http://onlinelibrary.wiley.com/doi/10.1002/14651858.CD007401.pub3> [non-English = 0 ; unpublished = 0 ; dissertations = 0]

5. Bukirwa H, Critchley Julia A. Sulfadoxine-pyrimethamine plus artesunate versus sulfadoxine-pyrimethamine plus amodiaquine for treating uncomplicated malaria. Cochrane Database of Systematic Reviews [Internet]. 2006; (1). Available from: <http://onlinelibrary.wiley.com/doi/10.1002/14651858.CD004966.pub2> [non-English = 0 ; unpublished = 0 ; dissertations = 0]

6. Christopher Prince RH, David Kirubah V, John Sushil M, Sankarapandian V. Antibiotic therapy for Shigella dysentery. Cochrane Database of Systematic Reviews [Internet]. 2010; (8). Available from: <http://onlinelibrary.wiley.com/doi/10.1002/14651858.CD006784.pub4> [non-English = 0 ; unpublished = 0 ; dissertations = 0]

7. Cuello-García Carlos A, Pérez-Gaxiola G, Jiménez Gutiérrez C. Treating BCG-induced disease in children. Cochrane Database of Systematic Reviews [Internet]. 2013; (1). Available from: <http://onlinelibrary.wiley.com/doi/10.1002/14651858.CD008300.pub2> [non-English = 0 ; unpublished = 0 ; dissertations = 0]

8. de Bruyn G, Hahn S, Borwick A. Antibiotic treatment for travellers' diarrhoea. Cochrane Database of Systematic Reviews [Internet]. 2000; (3). Available from: <http://onlinelibrary.wiley.com/doi/10.1002/14651858.CD002242> [non-English = 0 ; unpublished = 0 ; dissertations = 0]

9. Ejemot-Nwadiaro Regina I, Ehiri John E, Meremikwu Martin M, Critchley Julia A. Hand washing for preventing diarrhoea. Cochrane Database of Systematic Reviews [Internet]. 2008; (1). Available from: <http://onlinelibrary.wiley.com/doi/10.1002/14651858.CD004265.pub2> [non-English = 0 ; unpublished = 0 ; dissertations = 0]

10. Galappaththy Gawrie NL, Tharyan P, Kirubakaran R. Primaquine for preventing relapse in people with Plasmodium vivax malaria treated with chloroquine. Cochrane Database of Systematic Reviews [Internet]. 2013; (10). Available from: <http://onlinelibrary.wiley.com/doi/10.1002/14651858.CD004389.pub3> [non-English = 0; unpublished = 0 ; dissertations = 0]

11. Gonzales Maria Liza M, Dans Leonila F, Martinez Elizabeth G. Antiamoebic drugs for treating amoebic colitis. Cochrane Database of Systematic Reviews [Internet]. 2009; (2). Available from: <http://onlinelibrary.wiley.com/doi/10.1002/14651858.CD006085.pub2> [non-English = 0 ; unpublished = 0 ; dissertations = 0]

12. Graves Patricia M, Deeks Jonathan J, Demicheli V, Jefferson T. Vaccines for preventing cholera: killed whole cell or other subunit vaccines (injected). Cochrane Database of Systematic Reviews [Internet]. 2010; (8). Available from: <http://onlinelibrary.wiley.com/doi/10.1002/14651858.CD000974.pub2> [non-English = 0 ; unpublished = 0 ; dissertations = 0]

13. Graves Patricia M, Gelband H. Vaccines for preventing malaria (SPf66). Cochrane Database of Systematic Reviews [Internet]. 2006; (2). Available from: <http://onlinelibrary.wiley.com/doi/10.1002/14651858.CD005966> [non-English = 0 ; unpublished = 0 ; dissertations = 0]

14. Graves Patricia M, Gelband H. Vaccines for preventing malaria (pre-erythrocytic). Cochrane Database of Systematic Reviews [Internet]. 2006; (4). Available from: <http://onlinelibrary.wiley.com/doi/10.1002/14651858.CD006198> [non-English = 1 ; unpublished = 0 ; dissertations = 0]

15. Gregorio Germana V, Dans Leonila F, Silvestre Maria A. Early versus Delayed Refeeding for Children with Acute Diarrhoea. Cochrane Database of Systematic Reviews [Internet]. 2011; (7). Available from: <http://onlinelibrary.wiley.com/doi/10.1002/14651858.CD007296.pub2> [non-English = 0 ; unpublished = 0 ; dissertations = 0]

16. Hahn S, Kim Y, Garner P. Reduced osmolarity oral rehydration solution for treating dehydration caused by acute diarrhoea in children. Cochrane Database of Systematic Reviews [Internet]. 2002; (1). Available from: <http://onlinelibrary.wiley.com/doi/10.1002/14651858.CD002847> [non-English = 0 ; unpublished = 0 ; dissertations = 0]

17. Hartling L, Bellemare S, Wiebe N, Russell Kelly F, Klassen Terry P, Craig William R. Oral versus intravenous rehydration for treating dehydration due to gastroenteritis in children. Cochrane Database of Systematic Reviews [Internet]. 2006; (3). Available from: <http://onlinelibrary.wiley.com/doi/10.1002/14651858.CD004390.pub2> [non-English = 3 ; unpublished = 0 ; dissertations = 0]

18. Lazzerini M, Ronfani L. Oral zinc for treating diarrhoea in children. Cochrane Database of Systematic Reviews [Internet]. 2013; (1). Available from: <http://onlinelibrary.wiley.com/doi/10.1002/14651858.CD005436.pub4> [non-English = 0 ; unpublished = 0 ; dissertations = 0]

19. Lengeler C. Insecticide-treated bed nets and curtains for preventing malaria. Cochrane Database of Systematic Reviews [Internet]. 2004; (2). Available from: <http://onlinelibrary.wiley.com/doi/10.1002/14651858.CD000363.pub2> [non-English = 0 ; unpublished = 0 ; dissertations = 0]

20. Lesi Afolabi FE, Meremikwu Martin M. High first dose quinine regimen for treating severe malaria. Cochrane Database of Systematic Reviews [Internet]. 2004; (3). Available from: <http://onlinelibrary.wiley.com/doi/10.1002/14651858.CD003341.pub2> [non-English = 1 ; unpublished = 0 ; dissertations = 0]

21. Lutge Elizabeth E, Wiysonge Charles S, Knight Stephen E, Volmink J. Material incentives and enablers in the management of tuberculosis. Cochrane Database of Systematic Reviews [Internet]. 2012; (1). Available from: <http://onlinelibrary.wiley.com/doi/10.1002/14651858.CD007952.pub2> [non-English = 0 ; unpublished = 0 ; dissertations = 0]

22. Mayosi Bongani M. Interventions for treating tuberculous pericarditis. Cochrane Database of Systematic Reviews [Internet]. 2002; (4). Available from: <http://onlinelibrary.wiley.com/doi/10.1002/14651858.CD000526> [non-English = 0 ; unpublished = 0 ; dissertations = 0]

23. McIntosh H, Olliaro P. Artemisinin derivatives for treating uncomplicated malaria. Cochrane Database of Systematic Reviews [Internet]. 1999; (2). Available from: <http://onlinelibrary.wiley.com/doi/10.1002/14651858.CD000256> [non-English = 0 ; unpublished = 0 ; dissertations = 0]

24. McIntosh H, Olliaro P. Artemisinin derivatives for treating severe malaria. Cochrane Database of Systematic Reviews [Internet]. 2000; (2). Available from: <http://onlinelibrary.wiley.com/doi/10.1002/14651858.CD000527> [non-English = 0 ; unpublished = 0 ; dissertations = 0]

25. Meremikwu Martin M, Oyo-Ita A. Physical methods versus drug placebo or no treatment for managing fever in children. Cochrane Database of Systematic Reviews [Internet]. 2003; (2). Available from: <http://onlinelibrary.wiley.com/doi/10.1002/14651858.CD004264> [non-English = 0 ; unpublished = 0 ; dissertations = 0]

26. Meremikwu Martin M, Smith Helen J. Blood transfusion for treating malarial anaemia. Cochrane Database of Systematic Reviews [Internet]. 1999; (4). Available from: <http://onlinelibrary.wiley.com/doi/10.1002/14651858.CD001475> [non-English = 0 ; unpublished = 0 ; dissertations = 0]

27. Musekiwa A, Volmink J. Oral rehydration salt solution for treating cholera: 270 mOsm/L solutions vs 310 mOsm/L solutions. Cochrane Database of Systematic Reviews [Internet]. 2011; (12). Available from: <http://onlinelibrary.wiley.com/doi/10.1002/14651858.CD003754.pub3> [non-English = 0 ; unpublished = 0 ; dissertations = 0]

28. Okebe Joseph U, Yahav D, Shbita R, Paul M. Oral iron supplements for children in malaria-endemic areas. Cochrane Database of Systematic Reviews [Internet]. 2011; (10). Available from: <http://onlinelibrary.wiley.com/doi/10.1002/14651858.CD006589.pub3> [non-English = 0 ; unpublished = 0 ; dissertations = 2]

29. Onwuezobe Ifeanyi A, Oshun Philip O, Odigwe Chibuzo C. Antimicrobials for treating symptomatic non-typhoidal Salmonella infection. Cochrane Database of Systematic Reviews [Internet]. 2012; (11). Available from: <http://onlinelibrary.wiley.com/doi/10.1002/14651858.CD001167.pub2> [non-English = 0 ; unpublished = 0 ; dissertations = 0]

30. Panpanich R, Sornchai P, Kanjanaratanakorn K. Corticosteroids for treating dengue shock syndrome. Cochrane Database of Systematic Reviews [Internet]. 2006; (3). Available from: <http://onlinelibrary.wiley.com/doi/10.1002/14651858.CD003488.pub2> [non-English = 0 ; unpublished = 0 ; dissertations = 0]

31. Prasad K, Garner P. Steroids for treating cerebral malaria. Cochrane Database of Systematic Reviews [Internet]. 1999; (3). Available from: <http://onlinelibrary.wiley.com/doi/10.1002/14651858.CD000972> [non-English = 0 ; unpublished = 0 ; dissertations = 0]

32. Prasad K, Singh Mamta B. Corticosteroids for managing tuberculous meningitis. Cochrane Database of Systematic Reviews [Internet]. 2008; (1). Available from: <http://onlinelibrary.wiley.com/doi/10.1002/14651858.CD002244.pub3> [non-English = 0 ; unpublished = 0 ; dissertations = 0]

33. Sinclair D, Abba K, Zaman K, Qadri F, Graves Patricia M. Oral vaccines for preventing cholera. Cochrane Database of Systematic Reviews [Internet]. 2011; (3). Available from: <http://onlinelibrary.wiley.com/doi/10.1002/14651858.CD008603.pub2> [non-English = 0 ; unpublished = 0 ; dissertations = 0]

34. Sinclair D, Donegan S, Isba R, Lalloo David G. Artesunate versus quinine for treating severe malaria. Cochrane Database of Systematic Reviews [Internet]. 2012; (6). Available from: <http://onlinelibrary.wiley.com/doi/10.1002/14651858.CD005967.pub4> [non-English = 0 ; unpublished = 2 ; dissertations = 0]

35. Smith Helen J, Meremikwu Martin M. Iron-chelating agents for treating malaria. Cochrane Database of Systematic Reviews [Internet]. 2003; (2). Available from: <http://onlinelibrary.wiley.com/doi/10.1002/14651858.CD001474> [non-English = 0 ; unpublished = 0 ; dissertations = 0]

36. Soares-Weiser K, MacLehose H, Bergman H, Ben-Aharon I, Nagpal S, Goldberg E, et al. Vaccines for preventing rotavirus diarrhoea: vaccines in use. Cochrane Database of Systematic Reviews [Internet]. 2012; (11). Available from: <http://onlinelibrary.wiley.com/doi/10.1002/14651858.CD008521.pub3> [non-English = 0 ; unpublished = 0 ; dissertations = 0]

37. Taylor-Robinson David C, Maayan N, Soares-Weiser K, Donegan S, Garner P. Deworming drugs for soil-transmitted intestinal worms in children: effects on nutritional indicators, haemoglobin and school performance. Cochrane Database of Systematic Reviews [Internet]. 2012; (11). Available from: <http://onlinelibrary.wiley.com/doi/10.1002/14651858.CD000371.pub5> [non-English = 0 ; unpublished = 0 ; dissertations = 0]

38. Yousefi-Nooraie R, Mortaz-Hejri S, Mehrani M, Sadeghipour P. Antibiotics for treating human brucellosis. Cochrane Database of Systematic Reviews [Internet]. 2012; (10). Available from: <http://onlinelibrary.wiley.com/doi/10.1002/14651858.CD007179.pub2> [non-English = 0 ; unpublished = 0 ; dissertations = 0]

**34 systematic reviews with a least one meta-analysis from the Developmental Psychosocial and Learning Problems (DPLP) Cochrane Review Group**

1. Armelius B-Å, Andreassen Tore H. Cognitive-behavioral treatment for antisocial behavior in youth in residential treatment. Cochrane Database of Systematic Reviews [Internet]. 2007; (4). Available from: <http://onlinelibrary.wiley.com/doi/10.1002/14651858.CD005650.pub2> [non-English = 0 ; unpublished = 0 ; dissertations = 1]

2. Cheuk Daniel KL, Wong V, Chen Wen X. Acupuncture for autism spectrum disorders (ASD). Cochrane Database of Systematic Reviews [Internet]. 2011; (9). Available from: <http://onlinelibrary.wiley.com/doi/10.1002/14651858.CD007849.pub2> [non-English = 0 ; unpublished = 0 ; dissertations = 0]

3. Ching H, Pringsheim T. Aripiprazole for autism spectrum disorders (ASD). Cochrane Database of Systematic Reviews [Internet]. 2012; (5). Available from: <http://onlinelibrary.wiley.com/doi/10.1002/14651858.CD009043.pub2> [non-English = 0 ; unpublished = 0 ; dissertations = 0]

4. De-Regil Luz M, Jefferds Maria Elena D, Sylvetsky Allison C, Dowswell T. Intermittent iron supplementation for improving nutrition and development in children under 12 years of age. Cochrane Database of Systematic Reviews [Internet]. 2011; (12). Available from: <http://onlinelibrary.wiley.com/doi/10.1002/14651858.CD009085.pub2> [non-English = 0 ; unpublished = 0 ; dissertations = 1]

5. De-Regil Luz M, Suchdev Parminder S, Vist Gunn E, Walleser S, Peña-Rosas Juan P. Home fortification of foods with multiple micronutrient powders for health and nutrition in children under two years of age. Cochrane Database of Systematic Reviews [Internet]. 2011; (9). Available from: <http://onlinelibrary.wiley.com/doi/10.1002/14651858.CD008959.pub2> [non-English = 0 ; unpublished = 0 ; dissertations = 0]

6. Fernández-Gaxiola Ana C, De-Regil Luz M. Intermittent iron supplementation for reducing anaemia and its associated impairments in menstruating women. Cochrane Database of Systematic Reviews [Internet]. 2011; (12). Available from: <http://onlinelibrary.wiley.com/doi/10.1002/14651858.CD009218.pub2> [non-English = 0 ; unpublished = 0 ; dissertations = 0]

7. Gillies D, Sinn John KH, Lad Sagar S, Leach Matthew J, Ross Melissa J. Polyunsaturated fatty acids (PUFA) for attention deficit hyperactivity disorder (ADHD) in children and adolescents. Cochrane Database of Systematic Reviews [Internet]. 2012; (7). Available from: <http://onlinelibrary.wiley.com/doi/10.1002/14651858.CD007986.pub2> [non-English = 0 ; unpublished = 0 ; dissertations = 0]

8. Gold C, Wigram T, Elefant C. Music therapy for autistic spectrum disorder. Cochrane Database of Systematic Reviews [Internet]. 2006; (2). Available from: <http://onlinelibrary.wiley.com/doi/10.1002/14651858.CD004381.pub2> [non-English = 0 ; unpublished = 0 ; dissertations = 1]

9. Heirs M, Dean Mike E. Homeopathy for attention deficit/hyperactivity disorder or hyperkinetic disorder. Cochrane Database of Systematic Reviews [Internet]. 2007; (4). Available from: <http://onlinelibrary.wiley.com/doi/10.1002/14651858.CD005648.pub2> [non-English = 0 ; unpublished = 0 ; dissertations = 0]

10. Huband N, Ferriter M, Nathan R, Jones H. Antiepileptics for aggression and associated impulsivity. Cochrane Database of Systematic Reviews [Internet]. 2010; (2). Available from: <http://onlinelibrary.wiley.com/doi/10.1002/14651858.CD003499.pub3> [non-English = 0 ; unpublished = 0 ; dissertations = 0]

11. Huertas-Ceballos Angela A, Logan S, Bennett C, Macarthur C. Dietary interventions for recurrent abdominal pain (RAP) and irritable bowel syndrome (IBS) in childhood. Cochrane Database of Systematic Reviews [Internet]. 2009; (1). Available from: <http://onlinelibrary.wiley.com/doi/10.1002/14651858.CD003019.pub3> [non-English = 0 ; unpublished = 0 ; dissertations = 0]

12. Imdad A, Herzer K, Mayo-Wilson E, Yakoob Mohammad Y, Bhutta Zulfiqar A. Vitamin A supplementation for preventing morbidity and mortality in children from 6 months to 5 years of age. Cochrane Database of Systematic Reviews [Internet]. 2010; (12). Available from: <http://onlinelibrary.wiley.com/doi/10.1002/14651858.CD008524.pub2> [non-English = 0 ; unpublished = 0 ; dissertations = 0]

13. James S, Montgomery P, Williams K. Omega-3 fatty acids supplementation for autism spectrum disorders (ASD). Cochrane Database of Systematic Reviews [Internet]. 2011; (11). Available from: <http://onlinelibrary.wiley.com/doi/10.1002/14651858.CD007992.pub2> [non-English = 0 ; unpublished = 0 ; dissertations = 0]

14. Jesner Ora S, Aref-Adib M, Coren E. Risperidone for autism spectrum disorder. Cochrane Database of Systematic Reviews [Internet]. 2007; (1). Available from: <http://onlinelibrary.wiley.com/doi/10.1002/14651858.CD005040.pub2> [non-English = 0 ; unpublished = 0 ; dissertations = 0]

15. Kristjansson B, Petticrew M, MacDonald B, Krasevec J, Janzen L, Greenhalgh T, et al. School feeding for improving the physical and psychosocial health of disadvantaged students. Cochrane Database of Systematic Reviews [Internet]. 2007; (1). Available from: <http://onlinelibrary.wiley.com/doi/10.1002/14651858.CD004676.pub2> [non-English = 0 ; unpublished = 0 ; dissertations = 0]

16. Littell Julia H, Campbell M, Green S, Toews B. Multisystemic Therapy for social, emotional, and behavioral problems in youth aged 10-17. Cochrane Database of Systematic Reviews [Internet]. 2005; (4). Available from: <http://onlinelibrary.wiley.com/doi/10.1002/14651858.CD004797.pub4> [non-English = 0 ; unpublished = 0 ; dissertations = 0]

17. Livingstone N, Macdonald G, Carr N. Restorative justice conferencing for reducing recidivism in young offenders (aged 7 to 21). Cochrane Database of Systematic Reviews [Internet]. 2013; (2). Available from: <http://onlinelibrary.wiley.com/doi/10.1002/14651858.CD008898.pub2> [non-English = 0 ; unpublished = 0 ; dissertations = 0]

18. Lucas P, McIntosh K, Petticrew M, Roberts Helen M, Shiell A. Financial benefits for child health and well-being in low income or socially disadvantaged families in developed world countries. Cochrane Database of Systematic Reviews [Internet]. 2008; (2). Available from: <http://onlinelibrary.wiley.com/doi/10.1002/14651858.CD006358.pub2> [non-English = 0 ; unpublished = 0 ; dissertations = 0]

19. McArthur G, Eve Philippa M, Jones K, Banales E, Kohnen S, Anandakumar T, et al. Phonics training for English-speaking poor readers. Cochrane Database of Systematic Reviews [Internet]. 2012; (12). Available from: <http://onlinelibrary.wiley.com/doi/10.1002/14651858.CD009115.pub2> [non-English = 0 ; unpublished = 0 ; dissertations = 1]

20. Miller S, Maguire Lisa K, Macdonald G. Home-based child development interventions for preschool children from socially disadvantaged families. Cochrane Database of Systematic Reviews [Internet]. 2011; (12). Available from: <http://onlinelibrary.wiley.com/doi/10.1002/14651858.CD008131.pub2> [non-English = 0 ; unpublished = 0 ; dissertations = 0]

21. Montgomery P, Bjornstad Gretchen J, Dennis Jane A. Media-based behavioural treatments for behavioural problems in children. Cochrane Database of Systematic Reviews [Internet]. 2006; (1). Available from: <http://onlinelibrary.wiley.com/doi/10.1002/14651858.CD002206.pub3> [non-English = 0 ; unpublished = 0 ; dissertations = 0]

22. Oono Inalegwu P, Honey Emma J, McConachie H. Parent-mediated early intervention for young children with autism spectrum disorders (ASD). Cochrane Database of Systematic Reviews [Internet]. 2013; (4). Available from: <http://onlinelibrary.wiley.com/doi/10.1002/14651858.CD009774.pub2> [non-English = 0 ; unpublished = 0 ; dissertations = 0]

23. Petrosino A, Turpin-Petrosino C, Hollis-Peel Meghan E, Lavenberg Julia G. 'Scared Straight' and other juvenile awareness programs for preventing juvenile delinquency. Cochrane Database of Systematic Reviews [Internet]. 2013; (4). Available from: <http://onlinelibrary.wiley.com/doi/10.1002/14651858.CD002796.pub2> [non-English = 0 ; unpublished = 0 ; dissertations = 1]

24. Reichow B, Barton Erin E, Boyd Brian A, Hume K. Early intensive behavioral intervention (EIBI) for young children with autism spectrum disorders (ASD). Cochrane Database of Systematic Reviews [Internet]. 2012; (10). Available from: <http://onlinelibrary.wiley.com/doi/10.1002/14651858.CD009260.pub2> [non-English = 0 ; unpublished = 0 ; dissertations = 0]

25. Reichow B, Steiner Amanda M, Volkmar F. Social skills groups for people aged 6 to 21 with autism spectrum disorders (ASD). Cochrane Database of Systematic Reviews [Internet]. 2012; (7). Available from: <http://onlinelibrary.wiley.com/doi/10.1002/14651858.CD008511.pub2> [non-English = 0 ; unpublished = 0 ; dissertations = 0]

26. Schoonees A, Lombard M, Musekiwa A, Nel E, Volmink J. Ready-to-use therapeutic food for home-based treatment of severe acute malnutrition in children from six months to five years of age. Cochrane Database of Systematic Reviews [Internet]. 2013; (6). Available from: <http://onlinelibrary.wiley.com/doi/10.1002/14651858.CD009000.pub2> [non-English = 0 ; unpublished = 0 ; dissertations = 0]

27. Sguassero Y, de Onis M, Bonotti Ana M, Carroli G. Community-based supplementary feeding for promoting the growth of children under five years of age in low and middle income countries. Cochrane Database of Systematic Reviews [Internet]. 2012; (6). Available from: <http://onlinelibrary.wiley.com/doi/10.1002/14651858.CD005039.pub3> [non-English = 0 ; unpublished = 0 ; dissertations = 0]

28. Wang B, Zhan S, Gong T, Lee L. Iron therapy for improving psychomotor development and cognitive function in children under the age of three with iron deficiency anaemia. Cochrane Database of Systematic Reviews [Internet]. 2013; (6). Available from: <http://onlinelibrary.wiley.com/doi/10.1002/14651858.CD001444.pub2> [non-English = 0 ; unpublished = 1 ; dissertations = 0]

29. Winokur M, Holtan A, Valentine D. Kinship care for the safety, permanency, and well-being of children removed from the home for maltreatment. Cochrane Database of Systematic Reviews [Internet]. 2009; (1). Available from: <http://onlinelibrary.wiley.com/doi/10.1002/14651858.CD006546.pub2> [non-English = 0 ; unpublished = 0 ; dissertations = 5]

30. Woolfenden S, Williams Katrina J, Peat J. Family and parenting interventions in children and adolescents with conduct disorder and delinquency aged 10-17. Cochrane Database of Systematic Reviews [Internet]. 2001; (2). Available from: <http://onlinelibrary.wiley.com/doi/10.1002/14651858.CD003015> [non-English = 0 ; unpublished = 0 ; dissertations = 0]

31. Yeoh B, Woolfenden S, Lanphear B, Ridley Greta F, Livingstone N. Household interventions for preventing domestic lead exposure in children. Cochrane Database of Systematic Reviews [Internet]. 2012; (4). Available from: <http://onlinelibrary.wiley.com/doi/10.1002/14651858.CD006047.pub3> [non-English = 0 ; unpublished = 0 ; dissertations = 1]

32. Zehetner Anthony A, Orr N, Buckmaster A, Williams K, Wheeler Danielle M. Iron supplementation for breath-holding attacks in children. Cochrane Database of Systematic Reviews [Internet]. 2010; (5). Available from: <http://onlinelibrary.wiley.com/doi/10.1002/14651858.CD008132.pub2> [non-English = 0 ; unpublished = 0 ; dissertations = 0]

33. Zoritch B, Roberts I, Oakley A. Day care for pre-school children. Cochrane Database of Systematic Reviews [Internet]. 2000; (3). Available from: <http://onlinelibrary.wiley.com/doi/10.1002/14651858.CD000564> [non-English = 0 ; unpublished = 0 ; dissertations = 0]

34. Zwi K, Woolfenden S, Wheeler Danielle M, O'Brien T, Tait P, Williams Katrina J. School-based education programmes for the prevention of child sexual abuse. Cochrane Database of Systematic Reviews [Internet]. 2007; (3). Available from: <http://onlinelibrary.wiley.com/doi/10.1002/14651858.CD004380.pub2> [non-English = 0 ; unpublished = 0 ; dissertations = 0]

**Summary of Cochrane reviews, by group, which included non-English language studies, unpublished studies and/or dissertations**

| **Review Group: ARI (n=57)** | **Non-English** | **Unpublished** | **Dissertations** |
| --- | --- | --- | --- |
| # of Studies | 28 | 11 | 2 |
| # of Reviews | 11 | 5 | 1 |

**11 ARI systematic reviews which included Non-English language trials**

1. Chalumeau M, Duijvestijn Yvonne CM. Acetylcysteine and carbocysteine for acute upper and lower respiratory tract infections in paediatric patients without chronic broncho-pulmonary disease. Cochrane Database of Systematic Reviews [Internet]. 2013; (5). Available from: [http://onlinelibrary.wiley.com/doi/10.1002/14651858.CD003124.pub4](http://onlinelibrary.wiley.com/doi/10.1002/14651858.CD003124.pub4%20) [non-English = 3 ; unpublished = 0 ; dissertations = 0]
2. Del-Rio-Navarro Blanca E, Espinosa-Rosales Francisco J, Flenady V, Sienra-Monge Juan JL. Immunostimulants for preventing respiratory tract infection in children. Cochrane Database of Systematic Reviews [Internet]. 2006; (4). Available from: [http://onlinelibrary.wiley.com/doi/10.1002/14651858.CD004974.pub2](http://onlinelibrary.wiley.com/doi/10.1002/14651858.CD004974.pub2%20) [non-English = 12 ; unpublished = 0 ; dissertations = 0]
3. Gadomski Anne M, Brower M. Bronchodilators for bronchiolitis. Cochrane Database of Systematic Reviews [Internet]. 2010; (12). Available from: [http://onlinelibrary.wiley.com/doi/10.1002/14651858.CD001266.pub3](http://onlinelibrary.wiley.com/doi/10.1002/14651858.CD001266) [non-English = 1 ; unpublished = 2 ; dissertations = 0]
4. Hartling L, Bialy Liza M, Vandermeer B, Tjosvold L, Johnson David W, Plint Amy C, et al. Epinephrine for bronchiolitis. Cochrane Database of Systematic Reviews [Internet]. 2011; (6). Available from: <http://onlinelibrary.wiley.com/doi/10.1002/14651858.CD003123.pub3> [non-English = 1 ; unpublished = 0 ; dissertations = 0]
5. Hemilä H, Chalker E. Vitamin C for preventing and treating the common cold. Cochrane Database of Systematic Reviews [Internet]. 2013; (1). Available from: <http://onlinelibrary.wiley.com/doi/10.1002/14651858.CD000980.pub4> [non-English = 3 ; unpublished = 2 ; dissertations = 2]
6. Jiang L, Deng L, Wu T. Chinese medicinal herbs for influenza. Cochrane Database of Systematic Reviews [Internet]. 2013; (3). Available from: <http://onlinelibrary.wiley.com/doi/10.1002/14651858.CD004559.pub4> [non-English = 2 ; unpublished = 0 ; dissertations = 0]
7. Kenealy T, Arroll B. Antibiotics for the common cold and acute purulent rhinitis. Cochrane Database of Systematic Reviews [Internet]. 2013; (6). Available from: <http://onlinelibrary.wiley.com/doi/10.1002/14651858.CD000247.pub3> [non-English = 1 ; unpublished = 0 ; dissertations = 0]
8. Laopaiboon M, Panpanich R, Lerttrakarnnon P. Azithromycin for acute lower respiratory tract infections. Cochrane Database of Systematic Reviews [Internet]. 2008; (1). Available from: <http://onlinelibrary.wiley.com/doi/10.1002/14651858.CD001954.pub3> [non-English = 1 ; unpublished = 0 ; dissertations = 0]
9. Russell Kelly F, Liang Y, O'Gorman K, Johnson David W, Klassen Terry P. Glucocorticoids for croup. Cochrane Database of Systematic Reviews [Internet]. 2011; (1). Available from: <http://onlinelibrary.wiley.com/doi/10.1002/14651858.CD001955.pub3> [non-English = 1 ; unpublished = 0 ; dissertations = 0]
10. Schuetz P, Müller B, Christ-Crain M, Stolz D, Tamm M, Bouadma L, et al. Procalcitonin to initiate or discontinue antibiotics in acute respiratory tract infections. Cochrane Database of Systematic Reviews [Internet]. 2012; (9). Available from: <http://onlinelibrary.wiley.com/doi/10.1002/14651858.CD007498.pub2> [non-English = 1 ; unpublished = 0 ; dissertations = 0]
11. Venekamp Roderick P, Thompson Matthew J, Hayward G, Heneghan Carl J, Del Mar Chris B, Perera R, et al. Systemic corticosteroids for acute sinusitis. Cochrane Database of Systematic Reviews [Internet]. 2011; (12). Available from: <http://onlinelibrary.wiley.com/doi/10.1002/14651858.CD008115.pub2> [non-English = 2 ; unpublished = 0 ; dissertations = 0]

**5 ARI systematic reviews which included unpublished trials**

1. Brouwer Matthijs C, McIntyre P, Prasad K, van de Beek D. Corticosteroids for acute bacterial meningitis. Cochrane Database of Systematic Reviews [Internet]. 2013; (6). Available from: <http://onlinelibrary.wiley.com/doi/10.1002/14651858.CD004405.pub4> [non-English = 0 ; unpublished = 1 ; dissertations = 0]
2. Gadomski Anne M, Brower M. Bronchodilators for bronchiolitis. Cochrane Database of Systematic Reviews [Internet]. 2010; (12). Available from: <http://onlinelibrary.wiley.com/doi/10.1002/14651858.CD001266.pub3> [non-English = 1 ; unpublished = 2 ; dissertations = 0]
3. Hemilä H, Chalker E. Vitamin C for preventing and treating the common cold. Cochrane Database of Systematic Reviews [Internet]. 2013; (1). Available from: <http://onlinelibrary.wiley.com/doi/10.1002/14651858.CD000980.pub4> [non-English = 3 ; unpublished = 2 ; dissertations = 2]
4. Jefferson T, Jones Mark A, Doshi P, Del Mar Chris B, Heneghan Carl J, Hama R, et al. Neuraminidase inhibitors for preventing and treating influenza in healthy adults and children. Cochrane Database of Systematic Reviews [Internet]. 2012; (1). Available from: <http://onlinelibrary.wiley.com/doi/10.1002/14651858.CD008965.pub3> [non-English = 0 ; unpublished = 5 ; dissertations = 0]
5. Wang K, Shun-Shin M, Gill P, Perera R, Harnden A. Neuraminidase inhibitors for preventing and treating influenza in children (published trials only). Cochrane Database of Systematic Reviews [Internet]. 2012; (4). Available from: <http://onlinelibrary.wiley.com/doi/10.1002/14651858.CD002744.pub4> [non-English = 0 ; unpublished = 1 ; dissertations = 0]

**1 ARI systematic review which included Dissertations**

1. Hemilä H, Chalker E. Vitamin C for preventing and treating the common cold. Cochrane Database of Systematic Reviews [Internet]. 2013; (1). Available from: <http://onlinelibrary.wiley.com/doi/10.1002/14651858.CD000980.pub4> [non-English = 3 ; unpublished = 2 ; dissertations = 2]

| **Review Group: ID (n=38)** | **Non-English** | **Unpublished** | **Dissertations** |
| --- | --- | --- | --- |
| # of Studies | 6 | 3 | 2 |
| # of Reviews | 4 | 2 | 1 |

**4 ID systematic reviews which included non-English language studies**

1. Allen Stephen J, Martinez Elizabeth G, Gregorio Germana V, Dans Leonila F. Probiotics for treating acute infectious diarrhoea. Cochrane Database of Systematic Reviews [Internet]. 2010; (11). Available from: <http://onlinelibrary.wiley.com/doi/10.1002/14651858.CD003048.pub3> [non-English = 1 ; unpublished = 1 ; dissertations = 0]
2. Graves Patricia M, Gelband H. Vaccines for preventing malaria (pre-erythrocytic). Cochrane Database of Systematic Reviews [Internet]. 2006; (4). Available from: <http://onlinelibrary.wiley.com/doi/10.1002/14651858.CD006198> [non-English = 1 ; unpublished = 0 ; dissertations = 0]
3. Hartling L, Bellemare S, Wiebe N, Russell Kelly F, Klassen Terry P, Craig William R. Oral versus intravenous rehydration for treating dehydration due to gastroenteritis in children. Cochrane Database of Systematic Reviews [Internet]. 2006; (3). Available from: <http://onlinelibrary.wiley.com/doi/10.1002/14651858.CD004390.pub2> [non-English = 3 ; unpublished = 0 ; dissertations = 0]
4. Lesi Afolabi FE, Meremikwu Martin M. High first dose quinine regimen for treating severe malaria. Cochrane Database of Systematic Reviews [Internet]. 2004; (3). Available from: <http://onlinelibrary.wiley.com/doi/10.1002/14651858.CD003341.pub2> [non-English = 1 ; unpublished = 0 ; dissertations = 0]

**2 ID systematic reviews which included unpublished studies**

1. Allen Stephen J, Martinez Elizabeth G, Gregorio Germana V, Dans Leonila F. Probiotics for treating acute infectious diarrhoea. Cochrane Database of Systematic Reviews [Internet]. 2010; (11). Available from: <http://onlinelibrary.wiley.com/doi/10.1002/14651858.CD003048.pub3> [non-English = 1 ; unpublished = 1 ; dissertations = 0]
2. Sinclair D, Donegan S, Isba R, Lalloo David G. Artesunate versus quinine for treating severe malaria. Cochrane Database of Systematic Reviews [Internet]. 2012; (6). Available from: <http://onlinelibrary.wiley.com/doi/10.1002/14651858.CD005967.pub4> [non-English = 0 ; unpublished = 2 ; dissertations = 0]

**1 ID systematic review which included dissertations**

1. Okebe Joseph U, Yahav D, Shbita R, Paul M. Oral iron supplements for children in malaria-endemic areas. Cochrane Database of Systematic Reviews [Internet]. 2011; (10). Available from: <http://onlinelibrary.wiley.com/doi/10.1002/14651858.CD006589.pub3> [non-English = 0 ; unpublished = 0 ; dissertations = 2]

| **Review Group: DPLP (n=34)** | **Non-English** | **Unpublished** | **Dissertations** |
| --- | --- | --- | --- |
| # of Studies | 0 | 1 | 11 |
| # of Reviews | 0 | 1 | 7 |

**1 DPLP systematic review which included unpublished studies**

1. Wang B, Zhan S, Gong T, Lee L. Iron therapy for improving psychomotor development and cognitive function in children under the age of three with iron deficiency anaemia. Cochrane Database of Systematic Reviews [Internet]. 2013; (6). Available from: <http://onlinelibrary.wiley.com/doi/10.1002/14651858.CD001444.pub2> [non-English = 0 ; unpublished = 1 ; dissertations = 0]

**7 DPLP systematic reviews which included dissertations**

1. Armelius B-Å, Andreassen Tore H. Cognitive-behavioral treatment for antisocial behavior in youth in residential treatment. Cochrane Database of Systematic Reviews [Internet]. 2007; (4). Available from: <http://onlinelibrary.wiley.com/doi/10.1002/14651858.CD005650.pub2> [non-English = 0 ; unpublished = 0 ; dissertations = 1]
2. De-Regil Luz M, Jefferds Maria Elena D, Sylvetsky Allison C, Dowswell T. Intermittent iron supplementation for improving nutrition and development in children under 12 years of age. Cochrane Database of Systematic Reviews [Internet]. 2011; (12). Available from: <http://onlinelibrary.wiley.com/doi/10.1002/14651858.CD009085.pub2> [non-English = 0 ; unpublished = 0 ; dissertations = 1]
3. Gold C, Wigram T, Elefant C. Music therapy for autistic spectrum disorder. Cochrane Database of Systematic Reviews [Internet]. 2006; (2). Available from: <http://onlinelibrary.wiley.com/doi/10.1002/14651858.CD004381.pub2> [non-English = 0 ; unpublished = 0 ; dissertations = 1]
4. McArthur G, Eve Philippa M, Jones K, Banales E, Kohnen S, Anandakumar T, et al. Phonics training for English-speaking poor readers. Cochrane Database of Systematic Reviews [Internet]. 2012; (12). Available from: <http://onlinelibrary.wiley.com/doi/10.1002/14651858.CD009115.pub2> [non-English = 0 ; unpublished = 0 ; dissertations = 1]
5. Petrosino A, Turpin-Petrosino C, Hollis-Peel Meghan E, Lavenberg Julia G. 'Scared Straight' and other juvenile awareness programs for preventing juvenile delinquency. Cochrane Database of Systematic Reviews [Internet]. 2013; (4). Available from: <http://onlinelibrary.wiley.com/doi/10.1002/14651858.CD002796.pub2> [non-English = 0 ; unpublished = 0 ; dissertations = 1]
6. Winokur M, Holtan A, Valentine D. Kinship care for the safety, permanency, and well-being of children removed from the home for maltreatment. Cochrane Database of Systematic Reviews [Internet]. 2009; (1). Available from: <http://onlinelibrary.wiley.com/doi/10.1002/14651858.CD006546.pub2> [non-English = 0 ; unpublished = 0 ; dissertations = 5]
7. Yeoh B, Woolfenden S, Lanphear B, Ridley Greta F, Livingstone N. Household interventions for preventing domestic lead exposure in children. Cochrane Database of Systematic Reviews [Internet]. 2012; (4). Available from: <http://onlinelibrary.wiley.com/doi/10.1002/14651858.CD006047.pub3> [non-English = 0 ; unpublished = 0 ; dissertations = 1]
